# Complementary Feeding: Recommendations for the Introduction of Allergenic Foods and Gluten in the Preterm Infant

**DOI:** 10.3390/nu13072477

**Published:** 2021-07-20

**Authors:** Federica Chiale, Elena Maggiora, Arianna Aceti, Nadia Liotto, Alessandra Coscia, Chiara Peila, Maria Elisabetta Baldassarre, Enrico Bertino, Francesco Cresi

**Affiliations:** 1 Neonatal Pathology and Neonatal Intensive Care Unit, Sant’Anna Hospital, City of Health and Science University Hospital of Turin, University of Turin, 10126 Turin, Italy; federica.chiale@gmail.com (F.C.); alessandra.coscia@unito.it (A.C.); peila.chiara@gmail.com (C.P.); enrico.bertino@unito.it (E.B.); francesco.cresi@unito.it (F.C.); 2 Neonatal Intensive Care Unit, AOU Bologna, Department of Medical and Surgical Sciences, University of Bologna, 40138 Bologna, Italy; arianna.aceti2@unibo.it; 3 Fondazione IRCCS Ca’ Granda Ospedale Maggiore Policlinico, Neonatal Intensive Care Unit, 20122 Milan, Italy; nadia.liotto@policlinico.mi.it; 4 Department of Human Oncology and Medical Sciences, University “Aldo Moro”, 70121 Bari, Italy; mariaelisabetta.baldassarre@uniba.it

**Keywords:** complementary feeding, weaning, preterm infants, allergenic foods, gluten, celiac disease, food allergy

## Abstract

Background: The aim of this systematic review is to analyze the available literature on the introduction of allergenic foods and gluten among preterm infants. Methods: A systematic review of published studies concerning the introduction of gluten and allergenic foods in preterm infants was performed on PubMed and on the Cochrane Library. Results: Of the 174 PubMed results, 15 papers were considered suitable for the review. A total of 83 records were identified through the Cochrane Library search; eight papers were included in the review. Additional papers were identified from the reference lists of included studies. A secondary search was conducted on the same databases to find recommendations and advice regarding healthy full-term infants that could be translated to preterm infants. Therefore, 59 additional papers were included in the review. Conclusions: Current guidelines for the introduction of solid food cannot be directly transposed to preterm infants. Further research is needed to provide evidence-based guidelines regarding weaning in preterm infants. To date, we can suggest that in preterm infants allergenic foods and gluten may be introduced when complementary feeding is started, any time after 4 months of corrected age, avoiding delayed introduction and irrespective of infants’ relative risk of developing allergy. Avoiding large amounts of gluten during the first few weeks after gluten introduction and during infancy is advised, despite limited evidence to support this recommendation.

## 1. Introduction

The period of complementary feeding (CF) represents a significant part of the 1000-day critical window [1]. 

There are no guidelines available regarding the age of initiation of CF in preterm infants (<37 weeks gestation) who are at risk of altered postnatal growth [2,3]. In 1994, the minimum body weight of 5 kg was selected as the main criteria to wean preterm infants on solid foods by the “Committee on Medical Aspects of Food and Nutrition” (COMA report [4]). Later, the period between 5 and 8 months of uncorrected age was suggested for the initiation of CF in preterm infants [5,6,7]. Of late, the Italian Society of Neonatology has published a systematic review suggesting CF between 5–8 months chronological age in infants aged 3 months corrected age (CA) if the required developmental skills are present [8]. CA is defined by American Academy of Pediatrics (AAP) as chronological age reduced by the number of weeks born before 40 weeks of gestation. Developmental signs of readiness include infants holding their heads up when sitting, showing interest in what others are eating and opening their mouths when food approaches [9].

None of the scientific societies working on nutrition in infancy, including the European Society of Pediatric Gastroenterology and Nutrition (ESPGHAN), AAP or the Canadian Pediatric Society (CPS), indicate the time of introduction of CF for preterm infants [10]. The lack of guidelines concerning this issue has led primary-care pediatricians and caregivers to several different approaches [11,12,13,14].

The introduction of allergenic foods and gluten has always been considered a challenge of CF in healthy full-term infants. In preterm infants, this is an issue of great concern. Given the absence of specific guidelines at present, the purpose of this paper is to review the available literature concerning the introduction of allergenic foods and gluten among preterm infants.

## 2. Materials and Methods

### Literature Search

A systematic review of published studies regarding the introduction of gluten and allergenic foods in preterm infants was performed. No limitation regarding year of publication was applied. 

The following databases were searched: PubMed (http://www.ncbi.nlm.nih.gov/pubmed/, accessed on 1 March 2021) and the Cochrane Library (https://www.cochranelibrary.com/advanced-search, accessed on 1 March 2021). Recommendations, reviews, study protocols for reviews and trials published up to 1 March 2021 were eligible.

The search was conducted by F.Ch. Pertinent studies were identified from the abstract, and the reference lists of the selected papers were searched for additional studies. The titles and abstracts of the articles were checked by two independent reviewers (F.Ch. and E.M.). Of the potentially relevant studies, the integral papers were gathered. Their eligibility for inclusion was independently evaluated by two reviewers (F.Ch. and E.M.).

## 3. Results

### Literature Search

Of the 174 PubMed results, 15 papers were deemed relevant for the review [7,8,15,16,17,18,19,20,21,22,23,24,25,26,27]. The Cochrane Library search produced 83 records: 62 Cochrane reviews, 6 Cochrane protocols and 15 Cochrane trials. Amongst these, one was a PubMed search duplicate [21]. After examining the full-texts: two reviews [28,29], one protocol [10] and five trials [21,30,31,32,33] were included in the review.

Searching through the reference lists of the included studies, other papers were also selected. Given the shortage of available literature regarding the introduction of allergenic foods and gluten in preterm infants, the search was extended to include papers on this same issue in full-term infants. This additional search was carried out on PubMed and the Cochrane Library: 64 papers were selected and included in the review [1,3,4,5,6,9,11,12,13,14,34,35,36,37,38,39,40,41,42,43,44,45,46,47,48,49,50,51,52,53,54,55,56,57,58,59,60,61,62,63,64,65,66,67,68,69,70,71,72,73,74,75,76,77,78,79,80,81,82,83,84,85,86,87].

The main characteristics of the included studies are reported in Supplementary Table S1.

## 4. Discussion

This review aimed to analyze the literature for data to provide specific recommendations for the preterm infant concerning the introduction of allergenic foods and gluten among preterm infants.

We found a paucity of quality studies aimed at preterm infants and decided to supplement our search with results from studies and guidelines for term infants. However, these data should be used with caution as the preterm infant is not fully comparable to the term infant, even when corrected for age. Intestinal motility and permeability, hormonal and immune systems and the microbiome of the preterm infant are different. All this makes it essential and urgent to continue research in this direction.

### 4.1. Allergenic Food Introduction

Preterm infants were considered to be at higher risk of developing food allergies due to increased gut permeability [34], increased exposure to foreign food proteins and early introduction of solid foods [3,5].

Low levels of antibodies to cow’s milk and gliadin were found in preterm infants [35,47]. A large epidemiological study found a lower incidence of eczema in preterm infants compared to full-term infants [37]. Others have either confirmed the same results [19,38,39,40] or found no difference [26,41,42,43]. Only fetal growth restriction could be associated with increased risk of atopic disease [44]; however, Liem et al. [45] reported no significantly increased risks for food allergy development for either prematurity or low birth weight in the 1995 Manitoba Birth Cohort in Canada. Kvenshagen et al. [43] found no difference in the prevalence of IgE-mediated food allergies in children with atopic dermatitis between infants born preterm and full-term. Gut permeability appears indeed to rapidly adapt after birth, regardless of gestational age or birth weight [46]. 

Some studies proposed that preterm infants are at increased risk of allergies [3,5], notably those with a strong family history of atopy, with no direct link to breastfeeding or the time of allergenic foods introduction [24]. Increased risk of wheezing is mostly due to lung damage in the neonatal period [48]. In 2004, the observational study conducted by Morgan et al. [18] revealed that preterm infants who had four or more solid foods introduced before 17 weeks CA, or who had any solid foods introduced before 10 weeks CA, had an increased risk of eczema development. Furthermore, it suggested that introducing solid food starting from 3 months CA also reduced the potentially increased risk of eczema development. In contrast to this finding, another paper by the same group [21] demonstrated no association between the age of introduction of CF (≤12 or >12 weeks, post-term age) and the incidence of eczema (21% of the preterm infants developed eczema by 9 months CA). Yrjänä et al. [17] studied the timing of CF introduction in a cohort of 664 preterm infants: Late preterm infants started CF at a median CA of 1.9 months, without having an increased incidence of food allergies or atopic dermatitis. These data support the theory that the gut-associated lymphoid tissue of preterm infants is ready to meet food proteins and to begin the maturation process within 3 to 6 months, regardless of gestational age.

The most common pediatric food allergies are reactions to cow’s milk, hen’s egg, soy, peanuts, tree nuts, wheat, fish and shellfish. Although the majority of children with milk or egg allergies will become tolerant, few are at risk of developing atopic disorders or respiratory allergic diseases [49]. Over the last few years, there has been increasing evidence to support the early introduction of potentially allergenic foods to enhance the development of oral tolerance [50]. Furthermore, low incidence rates of peanut allergies can be observed in countries in which the culinary culture is rich in peanuts, and they are commonly used in the weaning process [51]. These studies are extremely valuable, as there is a significant rise in food allergies in high-income countries, where the current recommendation is to restrict and delay exposure to such foods. The development of immuno-tolerance to an antigen might require both repeated exposure, possibly during an early critical window, and modulation of other dietary factors including breastfeeding [52].

As a result, the European Society of Pediatric Allergy and Clinical Immunology (EAACI) and the ESPGHAN have produced joint guidelines [15].

In 2014 the EAACI Guidelines were released, providing evidence-based recommendations for primary prevention of food allergies [53]. The AAP’s advice is to not limit the maternal diet throughout pregnancy and lactation [15]. All infants should be exclusively breastfed for the first 4–6 months of life [25,28,29,77]. Should this not be possible or should it not be in adequate measure, a hypoallergenic formula can be recommended to high-risk infants for the first 4 months [78], after which a standard cow’s milk-based formula is recommended. After 4 months of age, CF is recommended, also for infants with a hereditary predisposition to atopy. No special measures should be taken in high-risk infants, neither withholding nor exposing them to “highly allergenic” foods, during the weaning process [23,53]. 

In 2016, a systematic review and meta-analysis by Ierodiakonou [57] showed that timing of introduction of allergenic foods was associated with the risk of allergic disease. Introducing eggs at 4–6 months of age was associated with reduced rates of egg allergy across the allergy-risk spectrum, from normal risk to very high risk, in the infant population. Prior sensitization may cause severe allergic reactions in case of exposure to raw pasteurized eggs instead of cooked or heated eggs [52]. The same paper [57] also conducted observations regarding peanut allergies, and the conclusions overlap with those regarding egg introduction: The exposure to peanuts between 4 and 11 months of age is associated with fewer peanut allergies. This is the same conclusion the normal-risk population-based trial EAT [83] and the high-risk population-based trial LEAP [60] reached. The LEAP study defines high-risk infants for peanut allergies as those with severe eczema, egg allergy, or both. Hence, this population should undergo early peanut exposure, following evaluation by an appropriately trained specialist, as recommended by 10 international pediatric allergy associations [62]. Furthermore, it was observed that the prevalence of peanut allergy was not increased in the follow-up of children from the LEAP trial [61]. The meta-analysis by Ierodiakonou concluded that early fish introduction was associated with reduced allergic sensitization [57]. 

These data conflict with previous recommendations to delay the introduction of allergenic foods to the infant’s diet and suggests that current guidelines may need to be revised [53,58,59]. The EAT trial has proven that in normal-risk infant populations, the exposure to six different allergenic foods at a very early age, such as 3–4 months, was safe, with no evidence of adverse effects on breastfeeding [36]. The association between early allergenic food introduction and food allergy to the same food was limited to few studies, and it was only statistically significant for eggs and peanuts. The phenomenon of oral tolerance in humans has been demonstrated only recently [60,61]. Oral tolerance appears to be antigen-specific: Early introduction of one allergenic food does not influence the development of an allergy to a different allergenic food [57]. The findings may not be generalizable beyond food allergies mediated by IgE antibodies.

A systematic review of 14 studies by Larson [55] has put forward evidence stating that the delayed introduction, delayed being after 9 months of age, of potentially allergenic foods is associated with an increased risk of developing food allergies. Moreover, diets rich in fruits, vegetables and homemade foods seem to be associated with fewer food allergies [56]. 

In light of these discoveries and having reviewed the available evidence, in 2017, the ESPGHAN CoN provided new recommendations for the introduction of CF [52].

The formulation of distinct recommendations for breastfed and formula-fed infants is not advisable. In a recent review, Agostoni et al. [16] found no high-quality studies relating to the risk of food allergies among breastfed infants compared with bottle-fed infants. Continued breastfeeding is recommended together with the introduction of CF [52], due to the supposed biological effect of the immuno-competent molecules in breast milk rather than due to epidemiological evidence [16,20]. Small volumes of cows’ milk may be added to complementary foods, but it can be considered as the main drink only after 12 months of age [52]. Exposure to food allergens should be encouraged during CF, after 4 months of age. The postponement of this practice is not associated with a reduced risk of allergy, regardless of an atopy hereditary predisposition [52,53,54]. High-risk peanut allergy infants should have peanuts introduced between 4 and 11 months, following evaluation by an appropriately trained professional [52].

In February 2021, the update of the 2014 EAACI guideline was published [84], based on a systematic review by the same group, which included 46 studies [85,86]. Supplementation with regular cow’s milk formula in breastfed infants in the first week of life is not recommended [84]. They confirm the most effective age to introduce well-cooked egg and peanut in an age-appropriate form is from 4 to 6 months of life. In populations where there is a high prevalence of peanut allergy, the EAACI Task Force suggests introducing peanuts into the infant diet in an age-appropriate form as part of complementary feeding.

Eczema is considered the highest risk factor for developing IgE-mediated food allergy; however, children without risk factors still develop food allergy [9]. Guidance from the American Academy of Allergy, Asthma, and Immunology; American College of Allergy, Asthma, and Immunology and the Canadian Society for Allergy and Clinical Immunology [9] is in line with 2021 EAACI guidelines and addressed to all infants, irrespective of their relative risk of developing allergy. Screening before introduction is not required. Strongly consider encouraging either home introduction, or offering a supervised oral food challenge for any positive skin prick test or serum IgE result. Once peanut and egg are introduced, regular ingestion should be maintained [9]. 

Other allergens should be introduced around 6 months but not before 4 months as well. During introduction of CF, infants should be fed a diverse diet in order to help foster prevention of food allergy [9]. 

The 2021 EAACI guidelines try to answer the questions on the optimal timing and doses of food allergens that should be used to induce tolerance [84]. However, recommendations for preterm infants are still lacking. There are high costs involved in food allergies both for the healthcare system and society at large; therefore, it is reasonable to argue that research in primary prevention should be a priority, especially in preterm infants, a largely understudied population.

### 4.2. Gluten Introduction

Celiac disease (CD) is a chronic, multi-organ autoimmune disease that affects the small bowel in genetically predisposed persons precipitated by the ingestion of gluten. Based on analysis of the Norwegian MoBa cohort, the development of CD in children is not associated with intrauterine growth [65].

Prevention of the occurrence of CD with infant feeding practices has been extensively discussed. In 2008, the ESPGHAN CoN recommended avoiding both early (<4 months of age) and late (>7 months of age) gluten introduction. It also recommended introducing gluten while breastfeeding [66]. A recent meta-analysis showed that the introduction of gluten after 6 months increased the risk of developing CD [72].

Two recent RCTs [63,64] and the TEDDY study [80] examined the effect of the age of gluten introduction on the risk of developing CD autoimmunity (CDA) or CD in children at genetic risk for CD. While the incidence of early (<2 years old) CD and CDA was improved, the overall prevalence during childhood was not reduced. Therefore, changing the timing of the introduction of gluten cannot be considered a primary prevention measure. Furthermore, the lack of trustworthy data regarding the safety of very early (<3 months old) gluten introduction has discouraged such behavior [67].

Breastfeeding had no preventive effect on the development of CDA or CD during childhood according to the systematic review by Szajewska [67]. Only limited case-control evidence suggests that breastfeeding for short durations or not at all is associated with a higher risk of a diagnosis of IBD and celiac disease, respectively [76].

Despite the negative results of studies testing different strategies of the timing of gluten introduction, a high quantity of gluten remains a suggested risk factor [81]. Higher gluten intake during the first 5 years of life was related to the increased risk of CDA and CD in genetically predisposed children among participants in TEDDY [82]. However, this result was not confirmed by a re-evaluation of another large study of similarly at-risk children who developed CD [68]. Recognizing the absence of effective prevention strategies, the ESPGHAN published modified guidelines regarding gluten introduction [69,70,71]. 

Even though breastfeeding should be encouraged for its well-known health benefits, neither any breastfeeding nor breastfeeding during gluten introduction has been demonstrated to influence the risk of CD [69,75]. Introducing gluten while the infant is breastfed cannot be recommended to reduce the risk of developing CD [69]. The type of formula (cow’s milk protein or extensively hydrolyzed formulas) used to feed infants before 8 months of age did not affect the risk of developing CD [16].

Gluten may be introduced into the infant’s diet anytime between 4 and 12 months of age. The age of gluten introduction in infants in this age range does not seem to influence the absolute risk of developing CDA or CD during childhood [69]. There is still much to define regarding the introduction of gluten during CF. No advice can be given as to which types of gluten should be used during CF. 

ESPGHAN currently recommends avoiding large doses of gluten soon after its introduction and during infancy [52], despite limited evidence to support this recommendation. In the EAT Study [83], differently from other trials, gluten was introduced from age 4 months and in larger quantities: the findings from a prespecified analysis of the EAT trial indicate that early consumption of high-dose gluten should be considered as a strategy to prevent CD in future research [87].

No recommendation was made on gluten introduction in children from families with first-degree relatives with CD [69]. The very early development of CDA and CD (<3–5 years of age) seems to affect mainly children carrying the very-high-risk CD alleles (HLA-DQ2.5 homozygous), which are found in 1% to 2% of the general population but in 10% to 15% of children with first-degree relatives having CD. Earlier introduction of gluten (4–6 months) is associated with earlier development of CDA and CD in at-risk infants, although the cumulative incidence of both in later childhood is similar [14,66]. However, delaying the introduction of gluten may delay the onset of the disease, with potential benefit secondary to the maintenance of a state of health during a crucial period of child development [63].

It is well known that in children with no genetic predisposition for CD, the timing and mode of gluten introduction do not influence the risk. Though the recommendations are only valuable to genetically predisposed infants because they are the only ones at risk of developing CD after gluten introduction, the protocol must be applied to all infants, given that genetic risk alleles are generally not known at the time of introduction of CF [52,69]. Advice for preterm infants is extremely inadequate. 

## 5. Conclusions

The introduction of allergenic food and gluten is a major concern of CF. As we reported, the loss of gluten tolerance is a dynamic process; neither breastfeeding nor the delayed introduction are effective in preventing the later development of CD. Concerning food allergy prevention, the current paradigm is shifting from avoidance to controlled exposure, with increasing amounts of evidence to support the early introduction of potentially allergenic foods to aid in the development of oral tolerance.

To date, there is a severe shortage of evidence-based recommendations regarding CF in preterm infants [73], with CF being defined as the initiation of semisolid, soft or solid foods other than breast milk and follow-on formula [10]. Given the lack of univocal guidelines, primary-care pediatricians have developed different approaches in regard to CF in preterm infants, the most common approach being the avoidance of potentially allergenic foods, such as eggs, nuts, seeds and tomatoes [11,12]. According to King [5], it seems proper to apply the same strategies of healthy full-term infants to preterm infants. However, current guidelines for the introduction of solid food cannot be directly translated to preterm infants [15]: The high nutrient demands combined with the organ immaturity make it hard to reach the dietary intakes that will allow preterm infants to face their extrauterine growth. 

In light of the strict interrelationship between early nutrition and long-term health, specific recommendations on the optimal timing and strategy of complementary food introduction in preterm infants are needed, with a special focus on their specific nutritional requirements. Given the contrasting reports, RCTs specifically designed to assess the benefits and the risks of the age when and the way in which CF is started in preterm infants are needed [15]. Some RCTs on CF in preterm infants are ongoing [30,31,32,33], having atopic disease or food allergy as secondary outcomes. A protocol for a Cochrane Review (intervention) is now being evaluated by Gupta [10] with the aim of examining the initiation of CF in preterm infants, considering both corrected and postnatal age and evaluating the association with an increased risk of allergies and celiac disease. 

Having reviewed the literature and given the available evidence, our conclusions and recommendations regarding CF in preterm infants are that:

− “Highly allergenic” foods and gluten should be introduced during CF, after 4 months of corrected age, irrespective of infants’ relative risk of developing allergy. It is not recommended to delay or avoid such foods.
− Large amount of gluten are to be avoided soon after its introduction and during infancy, despite limited evidence to support this recommendation.

These conclusions are summarized in Supplementary Table S2.

Further research is needed to provide evidence-based guidelines regarding weaning preterm infants.

## Supplementary Materials

The following are available online at https://www.mdpi.com/article/10.3390/nu13072477/s1, Table S1: Main characteristics of the studies included in the review are described. Studies are classified according to study design: recommendations, reviews and study protocol for reviews, trials., Table S2: Summary of available evidence and our suggestion for introduction of allergenic foods and gluten in CF of preterm infants.
