# Supplementary material for: Complementary Feeding: Recommendations for the Introduction of Allergenic Foods and Gluten in the Preterm Infant
Source: Nutrients. 2021 Jul 20;13(7):2477. doi: 10.3390/nu13072477 (PMC8308791; doi:10.3390/nu13072477)
Supplement: Supplementary file 1 [file nutrients-13-02477-s001.zip › Table 1 Supplementary material REVISED.pdf]

**Table S1.** Main characteristics of the studies included in the review are described. Studies are classified according to study design: recommendations, reviews and study protocol for reviews, trials.

| *Author, year* | *Study design* | *Main findings* |
|---|---|---|
| **Recommendations** | | |
| COMA report, 1994 [4] | Recommendations | Report of the Working Group on the Weaning Diet of the Committee on Medical Aspects of Food Policy. Recommendations for CF in term infants. Brief specific advice for preterm infants. |
| Agostoni, 2008 [66] | Position paper | A Commentary by the ESPGHAN Committee on Nutrition on CF. It focuses on healthy infants in Europe. |
| King, 2009 [5] | Recommendations | Proposed evidence-based guide for CF tailored to preterm infants. |
| Fleischer, 2013 [59] | Recommendation | Recommendations from the Adverse Reactions to Foods Committee of the American Academy of Allergy, Asthma & Immunology for primary care physicians and specialists about the primary prevention of allergic disease through nutritional interventions according to current available literature and expert opinion. |
| Muraro, 2014 [53] | Guidelines | Prepared by the European Academy of Allergy and Clinical Immunology's (EAACI) Taskforce on Prevention and part of the EAACI Guidelines for Food Allergy and Anaphylaxis. It aims to provide evidence-based recommendations for primary prevention of food allergy. |
| Chan, 2014 [58] | Recommendation | The joint position statement of the Canadian Society of Allergy and Clinical Immunology and the Canadian Paediatric Society which reviews current evidence on dietary exposures and allergy prevention in infants at high risk of developing allergic conditions |
| Szajewska, 2016 [69] | Recommendation | ESPGHAN updated recommendations regarding gluten introduction in infants and the risk of developing coeliac disease during childhood. |
| Kumar, 2017 [6] | Consensus | Consensus summary on optimal nutrition to preterm LBW infants.<br>CF may be initiated at the corrected age of 4 months. No special attention is required while initiating CF. |

| | | |
|---|---|---|
| Fewtrell, 2017 [52] | Recommendation | A position paper from ESPGHAN Committee on Nutrition, which gives recommendations regarding CF focusing on healthy term infants in Europe. |
| EFSA, 2019 [7] | Recommendations | European Food Safety Authority (EFSA) Panel on Nutrition, Novel Foods and Food Allergens (NDA) discusses appropriate age range for introduction of CF into an infant's diet.<br>Advices for preterm infants: no evidence for an effect of introduction of CFs at 4 months post-term compared with 6 months post-term on body weight, body length and head circumference |
| Fleischer, 2020 [9] | Recommendation | A Consensus Approach to the Primary Prevention of Food Allergy Through Nutrition: Guidance from the American Academy of Allergy, Asthma, and Immunology; American College of Allergy, Asthma, and Immunology; and the Canadian Society for Allergy and Clinical Immunology. To help inform the primary care and allergy practicing clinician regarding the best current practices for infant feeding and food allergy prevention. |
| Halken, 2021 [84] | Guidelines | Update of the 2014 EAACI guideline to prevent the development of immediate-onset food allergy. |
| | **Reviews** | |
| Calder, 2006 [20] | Report | This report summarizes the content of the lectures of the Workshop held in February 2006 about recent findings in the areas of oral tolerance, routes of sensitization to allergens and factors affecting the development of atopic disease |
| Kramer, 2012 [28] | Cochrane review | Exclusive breastfeeding for six months versus exclusive breastfeeding for three to four months with mixed breastfeeding (introduction of complementary liquid or solid foods with continued breastfeeding) thereafter through six months. |
| Palmer, 2012 [3] | Narrative review | Available guidelines and current practices regarding PCF; evaluation of possible harms of early introduction of solid foods |
| Turck, 2013 [25] | Narrative review | Review on breastfeeding and health benefits for child and mother. |
| Krebs, 2014 [1] | Narrative review | Food-based strategies on outcomes related to micronutrient status, growth, and neurocognitive development |

| | | |
|---|---|---|
| De Silva, 2014 [78] | Systematic review | Ways to prevent the development of food allergy in children and adults |
| Szajewska, 2015 [67] | Systematic review with meta-analysis | Evidence on early feeding practices and the risk of coeliac disease |
| Fleischer, 2015 [62] | Interim guidance | Consensus communication on early peanut introduction and the prevention of peanut allergy in high-risk infants |
| Chmielewska, 2015 [75] | Narrative review | Impact of early feeding practices on the risk of developing CD during childhood. |
| Pinto-Sánchez, 2016 [72] | Systematic review and meta-analysis | Evidence regarding the effect of time of gluten introduction and breastfeeding on the risk of developing celiac disease |
| Ierodiakonou, 2016 [57] | Systematic review and meta-analysis | Association between early egg or peanut introduction to the infant diet and risk of developing egg or peanut allergy |
| Smith, 2016 [29] | Cochrane review | Benefits and harms of additional food or fluid for full-term healthy breastfeeding infant, examining the timing and type of additional food or fluid. |
| Embleton, 2017 [71] | Commentary on Gupta, 2017 | Commentary on the trial by Gupta et al. examining the effect of two different timing of PCF on preterm infants' growth and clinical outcome |
| West, 2017 [489 | Narrative review | Early introduction of allergenic foods for allergy prevention, focusing on the outcome of recent randomized controlled trials |
| Larson, 2017 [55] | Systematic review | Association between timing of introduction of potentially allergenic foods to infants and development of food allergies |
| Du Toit, 2017 [50] | Review | Evidence in support of early introduction of allergens into infant diets to prevent against the development of food allergy. |
| Barachetti, 2018 [15] | Narrative review | Management, timing and health outcomes of CF in preterm infants. |
| Peters, 2018 [27] | Narrative review | Nutrition after NICU discharge of preterm infants, with a focus on CF and allergenic foods |
| Vissers, 2018 [73] | Systematic review | Effect of the timing of CF introduction (early vs. late) on the risk of overweight in preterm infants |
| Lebwohl, 2018 [70] | Seminar | Seminar on celiac disease based on cohort and case-control studies, randomised trials, selected guidelines and systematic reviews and smaller, non-controlled clinical studies of particular relevance |

| | | |
|---|---|---|
| Gungor, 2019 [76] | Systematic review | Association between feeding human milk for short durations or not at all with risk of diagnosed IBD and celiac disease |
| Gungor, 2019 [77] | Systematic review | Association between feeding human milk for short durations or not at all with risk of childhood asthma, food allergies, allergic rhinitis, and atopic dermatitis. |
| Agostoni, 2019 [16] | Systematic review and meta-analysis | Health effects of different nutritional exposures or interventions in the first 2 y of life of healthy full-term infants in developed countries. |
| Liotto, 2020 [8] | Systematic review | Available literature regarding CF in preterm infants, with or without comorbidities that may interfere with oral functions |
| De Silva 2020 [86] | Systematic review | To inform EAACI guidelines. It found that most intervention might have little to no effect on preventing food allergy. |
| **Study protocol for reviews** | | |
| Gupta, 2016 [10] | Study protocol for a meta-analysis | To evaluate the effect and safety of early (at or before 4 months) vs. late (after 4 months) initiation of PCF. Both corrected and postnatal age are examined. |
| Vissers, 2016 [74] | Study protocol for Vissers, 2018 [72] | Protocol for a systematic review on the effect of PCF timing on overweight. |
| De Silva 2020 [85] | Systematic review protocol | Protocol for systematic review undertaken to hel prepare new EAACI guidelines. |
| **Trials** | | |
| Roberton, 1982 [34] | Prospective study | Forty-seven neonates born in three maternity hospitals were studied to determine absorption of β-lactoglobulin.<br>The results suggest that the ability of the gastrointestinal tract to exclude antigenically intact food proteins increases with gestational age. |
| Helms, 1987 [47] | Observational trial | Concentrations of IgG and IgM antibodies to casein, beta-lactoglobulin, lactalbumin and bovine serum albumin (BSA) in sera of premature infants of less than 36 weeks gestation, at 5 weeks of age, were less than in age-matched term infants. |
| David, 1988 [43[ | Observational trial | In a group of 443 children with atopic eczema there was a significant lack of subjects born before 37 weeks' gestation. |

| | | |
|---|---|---|
| Klebanoff, 1988 [41] | Observational trial | Data from prospective study Collaborative Perinatal Project with 44793 children who survived the first year and for whom the presence or absence of eczema was known. Preterm infants were at slightly decreased risk of eczema, but the difference was not significant. |
| De Martino, 1989 [42] | Observational trial | In 80 preterms aged 9-24 months and in 80 sex- and age-matched full-terms the frequency of atopic diseases and of positive skin tests to 8 food and 6 inhalant allergens was determined. Preterm infants fed human milk are not at increased risk of developing food allergy and related diseases |
| Lucas, 1990 [26] | Observational trials | Two randomised prospective trials.<br>Feeding neonates on formulas based on cows' milk, including those with a high protein content, did not increase the overall risk of allergy. Nevertheless, in the subgroup with a family history of atopy early exposure to cows' milk increased the risk of a wide range of allergic reactions, especially eczema. |
| Forster, 1990 [24] | Prospective study | Signs of atopy as well as interrelations with feeding regimens and family history of atopic disease were investigated at the age of 1 1/2 year. The study population was recruited from preterm and term babies.<br>No significant influence of breastfeeding, cow's milk formula and the time of intake of allergenic food on the clinical manifestation of atopy in any group. |
| Steffensen, 2000 [44] | Observational trial | Population of 4,795 male conscripts born between 1973 and 1975 in Denmark. Fetal growth retardation rather than preterm delivery of male infants is the main gestational factor underlying asthma and atopic dermatitis. |
| Siltanen, 2001 [39] | Observational trial | Two groups of 10-year-old children, 72 who were born preterm and 65 who were born at term. The children born preterm had significantly less atopy than the children born at term. |
| Hikino, 2001 [19] | Observational trial | Low birthweight was significantly associated with a lower risk of both food allergy and atopic dermatitis at 18 months of age |

| | | |
|---|---|---|
| Norris, 2002 [14] | Observational trial | Structured interviews conducted in the UK to evaluate factors associated with PCF.<br>Almost half of the infants received early CF, both considering corrected and chronological age. Differences between human milk- and formula-fed infants in the timing of CF were documented. |
| Siltanen, 2002 [35] | Observational trial | Early introduction of food antigens into the immature gastrointestinal tract of preterm infants might result in tolerance. The presence of less atopy in these children might also be a result of tolerance development. |
| Agosti, 2003 [40] | Observational trial | The incidence of allergic manifestations was evaluated from birth until 6 y of age in 83 very low birth weight infants (VLBWIs).<br>The incidence of total allergic manifestations (31.3%) in VLBWIs was significantly lower than that (52%) in 24-mo-old infants, born at full term. |
| Van Elburg, 2003 [46] | Observational trial | To determine the relation between intestinal permeability and birth weight, gestational age, postnatal age, and perinatal risk factors in neonates. In preterm infants (26–36 weeks gestation), intestinal permeability is not related to gestational age or birth weight |
| Siltanen, 2004 [48] | Observational trial | Two cohorts of 10-year-old children (72 children born preterm and 65 children born full-term). There was a significant difference between groups in the association of atopy with respiratory problems. |
| Morgan, 2004 [21] | Pooled RCTs results (Cochrane trial) | Data from >1600 term and preterm infants from five prospective UK RCTs, comparing early (<12 weeks) vs. late (>12 weeks) introduction of CF.<br>As for preterm infants, those weaned before 12 weeks showed slower gain in weight, length, and head circumference between 12 weeks and 18 months than those weaned after 12 weeks; by 18 months, there were no significant differences in size between the two groups. No effect of CF on other clinical outcomes was observed |
| Morgan, 2004 [18] | Observational trial | Evaluation of CF-related risk factors for eczema at 12 months post-term in preterm infants.<br>Identified risk factors were the introduction of ≥ 4 solid foods by or before 17 weeks post-term, male |

| | | |
|---|---|---|
| | | gender, having atopic parents who introduced solid foods before 10 weeks post-term or having at least one atopic parent. |
| Fanaro, 2007 [13] | Observational trial | Survey of CF practices in an Italian region. Wide variation in timing (corrected vs. chronological age) and quality of CF (low energy and low protein often offered as first solid food, with negligible iron and zinc content). |
| Liem, 2007 [45] | Observational trial | Evaluation of the 1995 Manitoba Birth Cohort in Canada.<br>No significantly increased risks were found for food allergy development from birth to 7 years of age for either prematurity or low birth weight. |
| Du Toit, 2008 [51] | Observational trial | Questionnaire-based study of 8600 schoolchildren. It demonstrated that Jewish children in the UK had a prevalence of peanut allergy that is 10-fold higher than that of Jewish children in Israel. |
| Kvenshagen, 2009 [43] | Observational trial | Prospective follow-up study over 2 years of 609 children (193 premature and 416 term). The prevalence of atopic dermatitis had no significant difference between preterm and term children |
| Nwaru, 2010 [54] | Observational trial | Data analysis from the Finnish Type 1 Diabetes Prediction and Prevention nutrition study, a prospective, birth cohort study.<br>Late introduction of solid foods was associated with increased risk of allergic sensitization to food and inhalant allergens |
| NCT01809548, 2013 [31] | RCT (Cochrane trial) | Prospective, randomized, two arm intervention study- the PIES project- to investigate the impact of different time points of introduction of complementary food on growth, body composition, atopic disease and neurodevelopmental outcome in preterm infant with a birth weight <1500 grams. |
| Grimshaw, 2013 [56] | Observational trial | Nested, case-control within a cohort study.<br>Infants who were diagnosed with food allergy by the time they were 2 years of age were introduced to solids earlier (#16 weeks of age) and were less likely to be receiving breast milk when cow's milk protein was first introduced into their diet |
| Lionetti, 2014 [63] | RCT | CELIPREV multicenter prospective study to evaluate the prevalence of celiac disease |

| | | |
|---|---|---|
| | | autoimmunity and of overt celiac disease among patients with a standard-risk or high-risk HLA genotype at 5 years of age. Neither the delayed introduction of gluten nor breastfeeding modified the risk of celiac disease among at-risk infants, although the later introduction of gluten was associated with a delayed onset of disease. A high-risk HLA genotype was an important predictor of disease |
| Vriezinga, 2014 [64] | RCT | A multicenter, randomized, double-blind, placebo-controlled dietary intervention study to evaluate the frequency of biopsy-confirmed celiac disease at 3 years of age (PreventCD). The introduction of small quantities of gluten at 16 to 24 weeks of age did not reduce the risk of celiac disease by 3 years of age in a group of high-risk children. |
| Emilsson, 2015 [65] | Prospective cohort study | Based on analysis of the Norwegian MoBa cohort, development of celiac disease in children is significantly associated with sex of the child, maternal celiac disease and type 1 diabetes, but not with intrauterine growth |
| Toro-Monjaraz, 2015 [23] | Observational trial | Retrospective, comparative, cross-sectional, observational study. The factors associated with cow's milk protein allergy were the use of antimicrobials during gestation and breastfeeding duration in months. No association was found with gestational age or type of delivery. |
| Aronsson, 2015 [80] | Observational trial | Multinational prospective observational birth cohort study (TEDDY The Environmental Determinants of Diabetes in the Young). The goal of this study was to determine whether age at introduction to gluten was associated with risk for celiac disease (CD) in genetically predisposed children.<br>In TEDDY, the time to first introduction to gluten introduction was not an independent risk factor for developing CD |
| CTRI/2015/11/006367, 2015 [32] | RCT (Cochrane trial) | Trial on preterm infants. Early weaning group (introduction of complementary foods at 4 months of chronological age) versus late weaning |

| | | |
|---|---|---|
| | | introduction of solid foods after 6 months of chronological age).<br>Any suspected or proven atopy, any wheezing episode requiring OPD visit/ hospital admissions as secondary outcome. |
| Du Toit, 2015 [60] | RCT | RCT with 640 infants with severe eczema, egg allergy, or both randomly assigned to consume or avoid peanuts until 60 months of age (LEAP). The early introduction of peanuts significantly decreased the frequency of the development of peanut allergy among children at high risk for this allergy and modulated immune responses to peanuts |
| Du Toit, 2016 [61] | RCT | Persistence of Oral Tolerance to Peanut (LEAP-On) study, which is a 12-month extension of the LEAP trial. Among children at high risk for allergy in whom peanuts had been introduced in the first year of life and continued until 5 years of age, a 12-month period of peanut avoidance was not associated with an increase in the prevalence of peanut allergy. |
| AndrénAronsson, 2016 [81] | Observational trial | 1-to-3 nested case-control study of 146 cases, resulting in 436 case-control pairs matched for sex, birth year, and HLA genotype generated from Swedish children at genetic risk for celiac disease.<br>The amount of gluten consumed until 2 years of age increases the risk of celiac disease at least 2-fold in genetically susceptible children. |
| Perkin, 2016 [83] | RCT | Exclusively breastfed infants 3 months old randomly assigned to the early introduction of six allergenic foods or to the current practice recommended in the United Kingdom of exclusive breastfeeding to approximately 6 months of age (EAT enquiring about tolerance study).<br>The trial did not show the efficacy of early introduction of allergenic foods in an intention-to-treat analysis. |
| Perkin, 2016 [36] | Observational trial | Feasibility of an early allergenic food introduction regimen in EAT study.<br>Early introduction, before 6 months of age, of at least some amount of multiple allergenic foods appears achievable and did not affect breastfeeding. |

| | | |
|---|---|---|
| Crespo-Escobar, 2017 [68] | Observational trial | Further analysis of the prospective PreventCD cohort study.<br>Gluten consumption patterns as well as the amount of gluten consumed at 11–36 months of age do not influence CD development for most related HLA genotypes in children with a genetic risk. This study reports the gluten consumption pattern in children at risk of CD from different European countries. |
| CTRI/2017/01/007702, 2017 [33] | RCT (Cochrane trial) | Trial on preterm infants. Early initiation of solid complementary feeds (between 11-13 weeks corrected age) versus delayed initiation of solid complementary feeds (between 15-17 weeks corrected age).<br>History of eczema as secondary outcome. |
| NCT03993860, 2017 [30] | RCT (Cochrane trial) | Single blinded randomized controlled trial, intervention group receiving fish powder versus control group receiving a placebo in the form of sorghum powder. Infants aged 6-months in a rural area in North-East Zambia.<br>Prevalence of fish allergy in the study area as secondary outcome |
| Baldassarre, 2018 [11] | Observational trial | Survey about PCF among Italian primary care pediatricians.<br>Heterogeneity in PCF timing (based on infant's age, and/or neurodevelopment and/or body weight), quality, and prescription of vitamin D and iron supplements. |
| Giannì, 2018 [12] | Observational trial | Evaluation of practices related to CF in a cohort of Italian late preterm infants.<br>Late preterm infants were weaned at almost 6 months of age and received low energy and/or low protein-dense foods as first solid foods. |
| Yrjänä, 2018 [17] | Observational trial | Evaluation of the association between very early introduction of semi-solid foods on food allergies or atopic dermatitis. Preterm infants were introduced safely to semi-solid foods earlier than term infants but did not show an increased risk for food allergies or atopic dermatitis. |
| AndrénAronsson, 2019 [82] | Observational trial | Multinational prospective observational birth cohort study (TEDDY The Environmental |

| | | |
|---|---|---|
| | | Determinants of Diabetes in the Young). The participants in TEDDY were followed up.<br>Higher gluten intake during the first 5 years of life was associated with increased risk of celiac disease autoimmunity and celiac disease among genetically predisposed children. |
| Logan 2020 [87] | Prespecified analysis of a RCT | Prespecified analysis of EAT RCT indicate that early consumption of high-dose gluten should be considered as a strategy to prevent CD in future research |
