# Supplementary material for: Complementary Feeding: Recommendations for the Introduction of Allergenic Foods and Gluten in the Preterm Infant
Source: Nutrients. 2021 Jul 20;13(7):2477. doi: 10.3390/nu13072477 (PMC8308791; doi:10.3390/nu13072477)
Supplement: Supplementary file 1 [file nutrients-13-02477-s001.zip › Table 2 Supplementary material.pdf]

Table S2. Summary of available evidence and our suggestion for introduction of allergenic foods and gluten in CF of preterm infants.

What do we know from papers in preterm infants	What we know from papers not in preterm infants	What we add to current guidelines
CF between the ages of 5-8 months chronological age in infants aged 3 months corrected age, if the required developmental skills are present *(strong evidence)*	Introduce food allergens in an age-appropriate form from 4 to 6 months of life, irrespective of infant's relative risk of developing allergy. Screening before introduction is not required *(strong evidence)*	"Highly allergenic" foods and gluten should be introduced during CF, after 4 months of corrected age, irrespective of infants' relative risk of developing allergy.
No increased risk of food allergy due only to preterm birth *(low evidence)*	Absence of effective prevention strategies for CD. Gluten may be introduced into the infant's diet anytime between 4 and 12 months of age *(strong evidence)*	Large amount of gluten are to be avoided soon after its introduction and during infancy, despite limited evidence to support this recommendation
No guidelines on introduction of food allergens and gluten in preterm infants.	Large amount of gluten are to be avoided soon after its introduction and during infancy, despite limited evidence to support this recommendation *(low evidence)*. However, early consumption of high-dose gluten should be considered as a strategy to prevent CD in future research.	
